# Orexin-A alleviates ferroptosis by activating the Nrf2/HO-1 signaling pathway in traumatic brain injury

**DOI:** 10.18632/aging.205541

**Published:** 2024-02-12

**Authors:** Junwei Kang, Bingkai Ren, Lianghua Huang, Xiaoyang Dong, Qi Xiong, Zhen Feng

**Affiliations:** 1 Department of Rehabilitation Medicine, First Affiliated Hospital of Nanchang University, Nanchang, Jiangxi 330006, PR China; 2 First Department of Rehabilitation Medicine, Affiliated Hospital of Jiangxi University of Traditional Chinese Medicine, Nanchang, Jiangxi 330006, China

**Keywords:** traumatic brain injury, ferroptosis, Orexin-A, oxidative stress

## Abstract

Background: Traumatic Brain Injury (TBI) has high disability and mortality rate. Oxidative stress and ferroptosis are important pathophysiological characteristics after TBI. Orexin-A (OXA) can alleviate neuronal damage in diverse neurological disorders. Nevertheless, the role and mechanism of OXA in TBI stay unknown.

Objectives: The research investigated protection influence of OXA on TBI and its potential mechanisms.

Methods: Male Sprague-Dawley rats were randomly grouped into: sham, TBI, TBI + normal saline (NS) and TBI+OXA groups. TBI model was constructed in rat via modified Feeney’s approach, and OXA treatment was administered following construction of TBI model.

Results: Relative to TBI+NS group, TBI+OXA group displayed greatly recovered tissue damage and neurological deficits. Additionally, OXA eased oxidative stress as well as ferroptosis in cerebral cortex of rats following TBI. Furthermore, OXA increased Nrf2 expression and regulating factors HO-1 and NQO1 in cerebral cortex of TBI rats.

Conclusions: Our research found OXA may restrain ferroptosis via Nrf2/HO-1 signaling pathway activation, thereby reducing brain injury after TBI.

## INTRODUCTION

Traumatic Brain Injury (TBI) is primary reason for trauma-related neurological dysfunction and death [1]. The annual number of patients worldwide suffering from TBI exceeds 50 million [2, 3]. TBI is mainly divided into primary and secondary injury. Primary injuries are neurological damages resulting from instantaneous external force, such as brain stem injury, diffuse axonal injury, and cerebral contusion and laceration, which is irreversible. Secondary injuries are continuous progressive nerve injuries that develop as a result of a series of pathophysiological changes after trauma, such as brain edema, cerebral ischemia, and neuron apoptosis, which can be alleviated or reversed by follow-up therapeutic intervention; therefore, secondary injury is the focus of clinical intervention [4]. The main pathological processes after TBI, in which clinical intervention is possible, include oxidative stress, inflammation, apoptosis, and ferroptosis. The mechanisms of some drugs have been preliminarily studied in the treatment of TBI; however, no particularly effective drugs have entered phase III clinical trials. Therefore, it is important to investigate new therapies that may reverse nerve injuries following TBI.

Research shows ferroptosis, vital for TBI development [5–8], is a new-found type of regulated cell death featuring iron-dependent lipid peroxidation. TBI leads to innumerable pathophysiological change, such as brain tissue destruction, blood-brain barrier collapse, cerebrovascular permeability increases and local severe inflammatory reactions, which result in vast iron efflux from blood into brain parenchyma, and can further cause excessive intracellular iron storage. Ferric iron is able to react with hydrogen peroxide through Fenton-like reaction for producing hydroxyl radical with stronger oxidation ability, increasing Reactive Oxygen Species (ROS) level in cell and inducing cell ferroptosis. The main biology characteristics of ferroptosis, cellular iron deposition and overload, excessive lipid peroxidation, reduction dysfunction, as well as disturbed redox homeostasis included, are important pathophysiological characteristics in the development of TBI. The ferroptosis inhibitor Fer-1 can reduce iron aggregation and neuron death in a model of TBI and recover motor and cognitive dysfunctions resulting from brain injury [9]. Thus, intervention against ferroptosis would be one possible approach for TBI treatment.

OXA, a neurotransmitter generated by lateral hypothalamus, participates in various brain activities, biological clock regulation, awakening, attention, emotion, movement, energy homeostasis, and cognition included [10–12]. Projecting from the lateral hypothalamus to diverse brain parts, cerebral cortex, brainstem and limbic system included, orexin neurons mediate activities from membrane excitation to cell death. OXA is highly lipophilic and can fastly pass through blood-brain barrier through an easy diffusion process [13]. Research done before found OXA exhibits neuroprotective influence in nervous system diseases like ischemic stroke and neurodegenerative disease through anti-inflammatory, anti-oxidation, and anti-apoptosis activity [14, 15]. According to findings from our previous research, electrical stimulation of nerves is able to amplify OXA secretion in lateral hypothalamus and alleviate brain injury via inflammation, oxidative stress and apoptosis inhibition. Furthermore, OXA is able to reduce inflammatory damages resulting from lipopolysaccharides (LPS) in neural stem cells [16–18]. Above discoveries greatly advise OXA would be a possible therapeutic compound for TBI. Currently, none reports about OXA influence on ferroptosis in TBI exist. Hence, for our research, whether OXA can alleviate nerve injury after TBI by alleviating ferroptosis and its potential mechanisms was initially investigated.

GPX4 plays a pivotal role in regulating cellular ferroptosis, while GSH, under the influence of GPX4, effectively mitigates the oxidation of phospholipids, thereby offering resistance against ferroptosis [19]. SLC7A11 serves as a dedicated amino acid transporter and a critical regulatory protein in ferroptosis [20]. The downregulation of SLC7A11 indirectly suppresses GPX4 activity by hindering the cysteine metabolism pathway. This leads to reduced intracellular cysteine levels, depletion of GSH biosynthesis, accumulation of lipid peroxides, and ultimately triggers cell ferroptosis.

Nuclear factor erythroid 2-related factor2 (NRF2) acts as a pivotal transcription factor governing cellular redox homeostasis and inflammation [21]. Activation of the Nrf2 signaling pathway also confers protection against ferroptosis. Furthermore, Nrf2, functioning as a transcription factor, upregulates the expression of both SLC7A11 and GPX4. Nrf2 governs multiple target genes, including proteins involved in intracellular redox balance such as heme oxygenase 1 (HO-1) and NADPH: quinone oxidoreductase 1 (NQO1). Hence, another facet of this study involves investigating whether OXA can enhance the neurological function of TBI-afflicted rats through the Nrf2/HO-1/NQO1 pathway.

## METHODS

### Reagents and materials

OXA was purchased from MedChemExpress (Monmouth Junction, NJ, USA), western blot and immunofluorescence primary antibodies from Abcam (Boston, MA, USA), and secondary antibodies from Proteintech (Wuhan, China), the malondialdehyde (MDA) content detection kit, superoxide dismutase (SOD) activity detection kit, and reduced glutathione (GSH) content detection kit from Solarbio Life Sciences (Beijing, China), Prussian blue staining kit from Servicebio (Wuhan, China), and hematoxylin and eosin (HE) staining kit from Leagene Biotechnology (Beijing, China).

### Animals

Animal experiments were all checked with approval from Ethics Committee of the First Affiliated Hospital of Nanchang University (Approval No. CDYFY-IACUC-202301QR057). Animal procedures were all performed strictly according to National Institutes of Health’s Guide for the Care and Use of Laboratory Animals. Specific Pathogen Free (SPF) male adult Sprague Dawley (SD) rats (6–8 weeks, weigh 250–300 g) supplied by Zhejiang Weitong Lihua Experimental Animal Technology Co., Ltd. (License No. SCXK (Zhejiang) 2019-0001) were selected for the experiment. Experimental animals were all placed in the same condition (room temperature 25°C, humidity 50%, 12-hour light/dark cycling) with water and food freely, and 7-day adaptation to the laboratory condition prior to the experiment.

### Construction of the TBI rat model and intracerebroventricular (ICV) injection

TBI rat model was constructed via a modified Feeney’s approach [22]. First, the animals experienced anesthesia with pentobarbital sodium (50 mg/kg, intraperitoneal injection) with their heads fixed in stereotactic frame, and a constant temperature heating pad was utilized for maintaining the body temperature. After disinfection of surgical area, a median incision (10 mm length) was created on scalp, and skin and periosteum were stripped. Then, at 3 mm posterior to coronal suture and 2.5 mm to the left of midline, one round bone window (5 mm diameter) was drilled with a cranial drill. The cranial disc was carefully removed without damaging the dura mater. Next, a T-shaped lance was placed outside the dura mater, and a 40 g weight was allowed to free-fall along the guide tube from 20 cm height to hit T-shaped lance, resulting in a compression depth of 2–3 mm without penetrating the dura mater, causing local brain contusion and laceration. Immediately after the impact, the T-shaped lance was removed from the injury position to avoid secondary injuries. The sham group experienced identical treatment as TBI group except for no impact on cerebral cortex. The stereotactic coordinates of the lateral ventricle were as follows: with the location of the bregma as the center, 1.5 mm to the side, 1.1 mm to the direction of posterior fontanelle, and a depth of 4.5 mm. After TBI modeling was completed, 10 μl of OXA (OXA dissolved in saline under concentration of 0.75 μg/μL, with an injection dose of 30 μg/kg) or 10 μL of 0.9% NaCl was injected with a speed of 2 μL/min.

### Experimental design

There were three parts to the experimental design of this study.

Part 1: First, we aimed at confirming expression trend of ferroptosis-associated molecules at diverse time points after TBI. The rats were haphazardly grouped into (*n* = 6 in each group): sham, 12 h, 24 h, 48 h and 72 h groups. Western blot was then performed.

Part 2: For exploring OXA influence on ferroptosis and neuronal damage after TBI in SD rats and its related pathways, rats were haphazardly grouped into (*n* = 6 in each group): sham, TBI, TBI+OXA, and TBI + normal saline (NS) groups. Western blot, real-time PCR, iron assay, Perls’ blue, Nissl, HE and FJB staining, immunohistochemistry, mNSS score test, were evaluated at indicated time-points following TBI.

Part 3: To further demonstrate that the biological effects of OXA depend on the Nrf2/HO-1 signaling pathway and to compare the effects of OXA and Lip-1, we used ML385 to inhibit the action of Nrf2. The rats were randomly divided into 5 groups (*n* = 6 in each group): TBI group, OXA group, Lip-1 group, OXA+ML385 group, Lip-1+ML385 group. Western blot and Hematoxylin-eosin (HE) staining were then performed.

After TBI modeling was completed, 10 μl of OXA (OXA dissolved in saline, with an injection dose of 30 μg/kg) was injected with a speed of 2 μL/min. 1 hour before modeling, the Nrf2 inhibitor ML385 (ML385 dissolved in DMSO, with an injection dose of 30 mg/kg) or equivalent volume of DMSO was injected intraperitoneally. 1 hour after TBI, the inhibitor of ferroptosis Lip-1 (Lip-1 dissolved in DMSO, with an injection dose of 10 mg/kg) or equivalent volume of DMSO was injected intraperitoneally.

### Quantitative polymerase chain reaction (qPCR)

Total RNA was extracted via TRizol reagent (Invitrogen, Carlsbad, CA, USA), and 800 ng of total RNA was reverse-transcribed into cDNA via Takara PrimeScript RT kit (Takara, Kyoto, Japan). Subsequently, qPCR was done via CFX96 Real-Time PCR Detection System. The mRNA levels were normalized to GAPDH to internal control. The mRNA expression was quantified using the 2^−ΔΔCt^ method. The calculation formula is as follows: ΔΔCt = ΔCt (experimental group) −ΔCt (control group); ΔCt (experimental group) = Ct (the target gene of the experimental group) −Ct (internal reference gene in the experimental group); ΔCt (control group) = Ct (the target gene of the control group) −Ct (internal reference gene in the control group); Fold Change = 2^−ΔΔCt^. We used one-way ANOVA to compare the statistical differences between the groups of samples. Primer sequence is detailed in Table 1.

**Table 1:** PCR primers utilized in our research.

**Gene**	**Accession number**	**Primer sequence (5′–3′)**		**Tm (°C)**	**CG%**	**Product size (bp)**
R-GAPDH	NM_017008.4	sense	AACAGCAACTCCCATTCTTCC	58.4	47.6	164
		antisense	TGGTCCAGGGTTTCTTACTCC	58.2	52.4	
R-GPX4	NM_001039849.3	sense	GAGGCAGGAGCCAGGAAGTA	59.3	60	213
		antisense	ACCACGCAGCCGTTCTTATC	60.1	55	
R-FACL4	NM_053623	sense	GAGCAAGGTTCAAGAGATGAATTAT	57.4	36	134
		antisense	GAGCCTTCACCTTCTTAAACAGTAT	58.8	40	
R-xCT	NM_001107673	sense	CTGTTATTGTTTTGCATCCTCTG	56.4	39.1	136
		antisense	TTGTATCGAAGATAAATCAGCCC	56.5	39.1	
R-FTH1	NM_012848.2	sense	TGAAGAACTTTGCCAAATACTTTC	58.1	33.3	164
		antisense	ACACTCCATTGCATTCAGCC	58.1	50	
R-COX2	NM_017232.3	sense	CATTCACCAGACAGATTGCTGG	60.4	50	137
		antisense	GAAGCGTTTGCGGTACTCATT	59.7	47.6	

### Biochemical assays

A lipid peroxidation, GSH, SOD, ROS, and tissue iron assay kits were used to evaluate the relative MDA, GSH, SOD, ROS, and iron ion concentrations in the cortex around the injury area, respectively. MDA levels were shown as nmol/mg protein, GSH levels as μMol/g protein, SOD levels as U/mg protein, ROS levels as fluorescence intensity/mg protein, and iron ion levels as μMol/g protein.

### Hematoxylin-eosin staining

Hematoxylin-eosin (HE) staining mainly observes the internal morphological structure of cells. The hematoxylin staining solution is alkaline, mainly staining the chromatin in the nucleus and the nucleic acids in the cytoplasm with purple-blue color. Eosin is an acidic dye, mainly staining the components of the cytoplasm and extracellular matrix with red color [23]. At 24 h after TBI, after completing behavioral testing, rats experienced anesthesia with pentobarbital sodium (50 mg/kg, intraperitoneal injection). Brain tissues were separated using cardiac perfusion followed by 24-hour fixing with 4% paraformaldehyde under ordinary temperature. After gradient dehydration with 75%, 95%, and 100% ethanol, the tissues were embedded in paraffin and cut into 5 μm-thick slices. Then, paraffin-embedded brain tissues experienced 6-minute and 3-minute staining by hematoxylin and eosin separately. Images were acquired via optical microscope.

### Nissl staining

Nissl body is distributed in the cytoplasm of neurons. Nissan contains RNA and is easily stained with alkaline dyes such as toluidine blue and tar violet [24]. Paraffin-embedded tissues were sliced into 5-μm-thick sections, experiencing dewaxing in xylene, rehydration in 100%, 95%, and 75% ethanol, 3-minute staining in toluidine blue and twice washing by distilled water (several seconds each time). After gradient dehydration with 75%, 95%, and 100% ethanol, slices experienced clearing in xylene and sealing by neutral resin before monitoring, imaging, and analysis via microscope.

### Perls staining

Perls stain, also known as the hemin stain, is a very classic histochemical reaction and a sensitive and traditional method for displaying trivalent iron in tissues. The principle of the staining process is that potassium ferrocyanide solution separates trivalent iron ions from proteins with dilute hydrochloric acid, and the trivalent iron reacts with potassium ferrocyanide to form an insoluble blue compound, namely trivalent iron ferrocyanide, also known as Prussian blue [25]. Paraffin-embedded slices experienced dewaxing and rehydration as section 2.8 and were subjected to Perls staining in the dark using a Prussian blue staining kit. The sections were sequentially incubated with the Perls working solution, incubation solution, and enhancing solution and washed thrice with distilled water. After dehydrating in gradient ethanol solutions, slices experienced clearing in xylene, sealing by neutral resin and observation via optical microscope.

### Immunohistochemistry

Paraffin slices were prepared, dewaxed, and rehydrated as in section 2.12 and experienced 30-minute immersing in preheated blocking permeating solution (40 ml PBS, 120 μl TritonX-100 and 400 μl 30% H_2_O_2_) (RT avoiding light) for reducing activity of endogenous peroxidase. Slices experienced thrice washing with PBS, immersing in 0.01 M sodium citrate buffer (pH 6.0) and 4-minute heating in microwave oven on high heat until boiling followed by being removed and cooled to room temperature which were repeated twice before slices experienced thrice washing by PBS. Slices experienced 30-minute incubation by normal goat serum under 37°C, and then incubation by anti-GPX4 (1:2000, Abcam, ab71495) and anti-prostaglandin peroxidase synthase 2 (PTGS2) (1:50, Abcam, ab9787) antibodies all night under 4°C. Following PBS washing, slices experienced 2-hour incubation by secondary antibody (goat anti-rabbit, 1:5,000, Zsgb Bio, China) under ordinary temperature. Images were acquired via optical microscope.

### Western blotting

After the behavioral assessment, the contusion cortex proteins around the injury edge were extracted (3 × 3 × 5 mm (width × deep × length)) and protein concentration was confirmed via Pierce BCA Protein Assay Kit (Thermo Fisher Scientific, Waltham, MA, USA). Protein levels of ferroptosis-associated proteins GPX4, PTGS2, SLC7A11, and pathway-related proteins HO-1, NQO1, and Nrf2 were determined using western blotting. The primary antibodies for western blotting included anti-GPX4 (1:1000, Abcam, ab32503), anti-PTGS2 (1:1000, Abcam, ab59348), anti-SLC7A11 (1:1000, Cell Signaling Technology, USA, 8242), anti-NRF2 (1:500, Abcam, ab214185), anti-HO-1 (1:500, Abcam, ab175449), anti-NQO1 (1:1000, Novusbio, USA, NB10056565), as well as anti-GAPDH (1:2000, Proteintech, 60004-1-lg). After sodium dodecyl sulfate-polyacrylamide gel electrophoresis, proteins of all samples were transferred to nitrocellulose filter membrane. For binding primary antibody to secondary antibody, membrane experienced incubation by secondary antibody horseradish peroxidase-labeled goat anti-rabbit or goat anti-mouse for 60 min under ordinary temperature. Western blot results were analyzed using enhanced chemiluminescence.

### Behavioral test

Before treatment with OXA and at 24 h after the injury, two experimentally blind individuals evaluated the rats using modified neurological severity score (mNSS). mNSS test owns ten diverse components to evaluate rats’ motor functions (muscle statuses and unusual movements), sensation (vision, touch, and proprioception), balance and reflex function. The score ranged from 0 to 18 (0, normal; 1–6, mild injury; 7–12, moderate injury; 13–18, serious damage; and 18, most severe neurological deficits).

### Statistical analysis

Continuous data (normal distribution) are shown as mean ± standard deviation. Great data difference among groups was confirmed through one-way analysis of variance with Tukey’s post hoc analysis. For mNSS data (non-normal distribution), great difference among groups was evaluated via Kruskal–Wallis test. Statistical analysis was done via GraphPad Prism 8.0 software. *P* < 0.05 was of statistical significance.

### Data availability statement

Data utilized for supporting research discoveries remain obtainable from corresponding author as required.

## RESULTS

### Changes in ferroptosis-associated proteins after TBI over time

**Figure 1:**
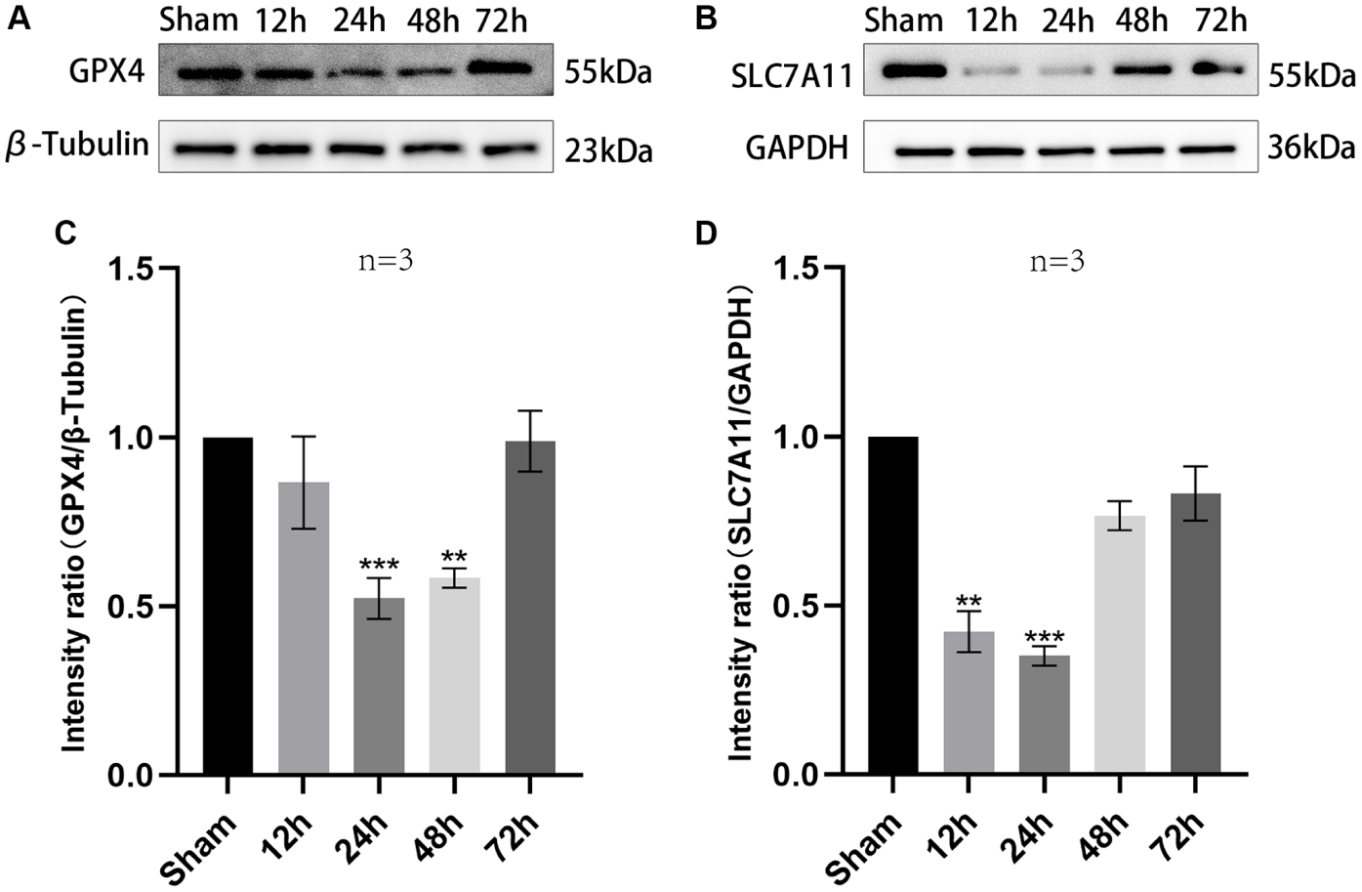
**The trend of expression of SLC7A11 and GPX4 in injured peripheral cortex of TBI rats over time.** (**A**, **B**) Representative WB bands of GPX4, SLC7A11, β-Tubulin and GAPDH in the injured peripheral cortex at a specified time point. (**C**, **D**) Comparison of relative expression levels of GPX4 and SLC7A11 in injured peripheral cortex of each rat group. Findings are shown as mean ± standard deviation. ^**^P < 0.01 vs. sham, ^***^P < 0.001 vs. sham.

For investigating ferroptosis function in pathophysiological process of TBI, expression levels of ferroptosis-associated proteins in the contused brain tissues around the edge of the injury at diverse time points were detected via western blotting. The expressions of SLC7A11 and GPX4 were analyzed. At 24 h after TBI, the overall trend of SLC7A11 and GPX4 decreased at first and then increased. It is worth noting that it was at the 24 h time point after TBI at which all these proteins showed statistical differences (GPX4: F (4,10) = 63.91, *P* < 0.001; SLC7A11: F (4,10) = 20.67, *P* < 0.001, Figure 1A–1D). Oxidative stress is an important pathophysiological process in the development of TBI. GPX4 can protect cell membranes and mitochondria from oxidative damage. Downregulation of SLC7A11 can indirectly inhibit the activity of GPX4 by inhibiting the cysteine metabolic pathway, leading to decreased intracellular cysteine levels and GSH biosynthesis depletion, resulting in accumulation of lipid peroxides and ultimately inducing cell death through ferroptosis. In this study, it was found that both GPX4 and SLC7A11 decreased at 24 hours after TBI, indicating that the degree of ferroptosis was most pronounced at this time and oxidative stress was most severe. Subsequently, as vascular spasm resolved, GPX4 and SLC7A11 began to rise.

### OXA attenuates TBI-induced ferroptosis

**Figure 2:**
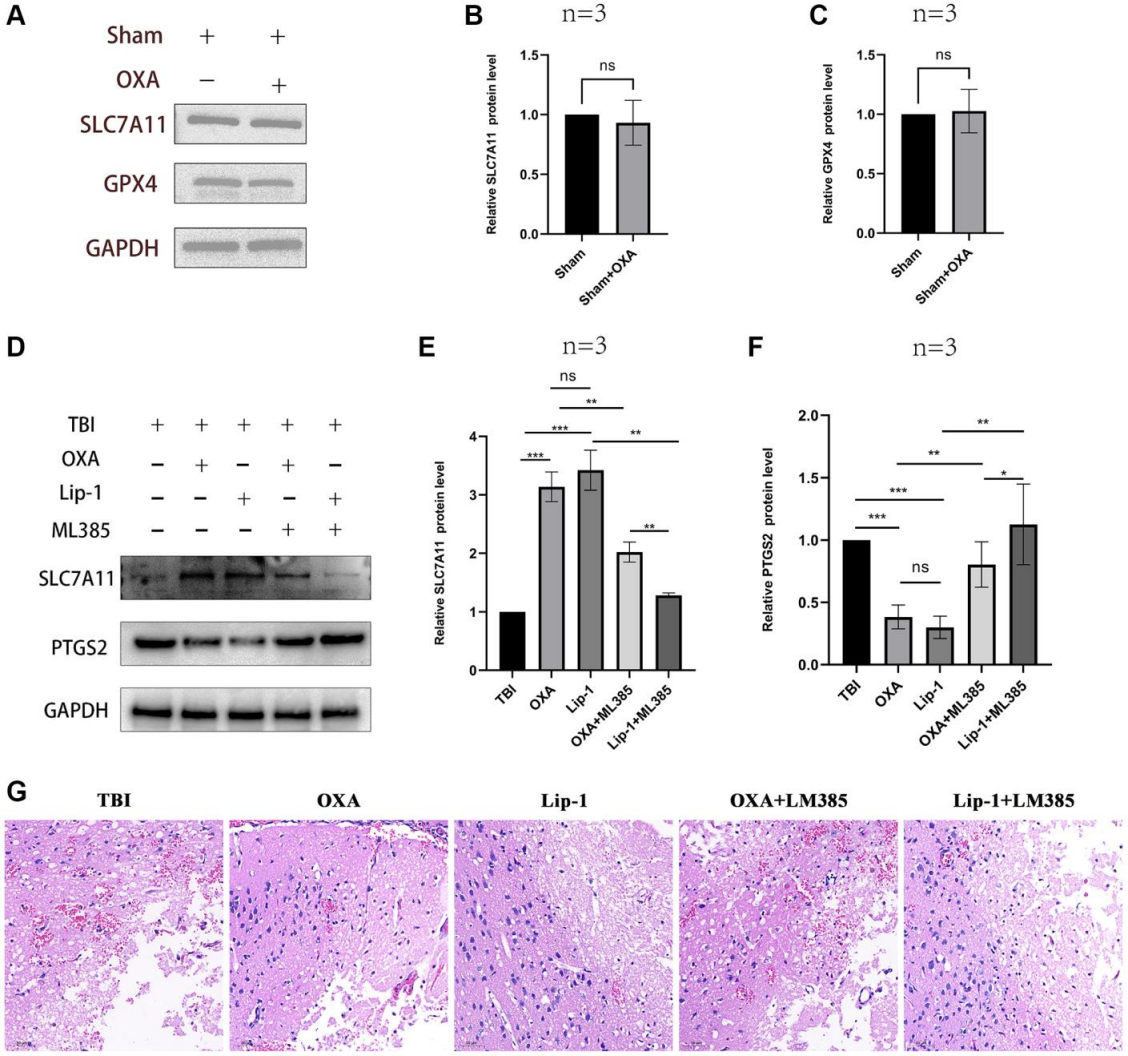
**The effect of OXA on the sham group rats and the effect of ML385 on OXA.** (**A**–**C**) The protein expression levels of GPX4 and SLC7A11 in sham group rats after intervention with OXA. (**D**–**F**) Comparison of the effects of OXA and Lip-1 on ferroptosis -related proteins SLC7A11 and PTGS2. (**G**) Typical HE staining images: comparison of the protective effects of OXA and Lip-1 on the brain. Statistical data are shown in mean ± SEM. ^*^P < 0.05, ^**^P < 0.01, ^***^P < 0.001.

**Figure 3:**
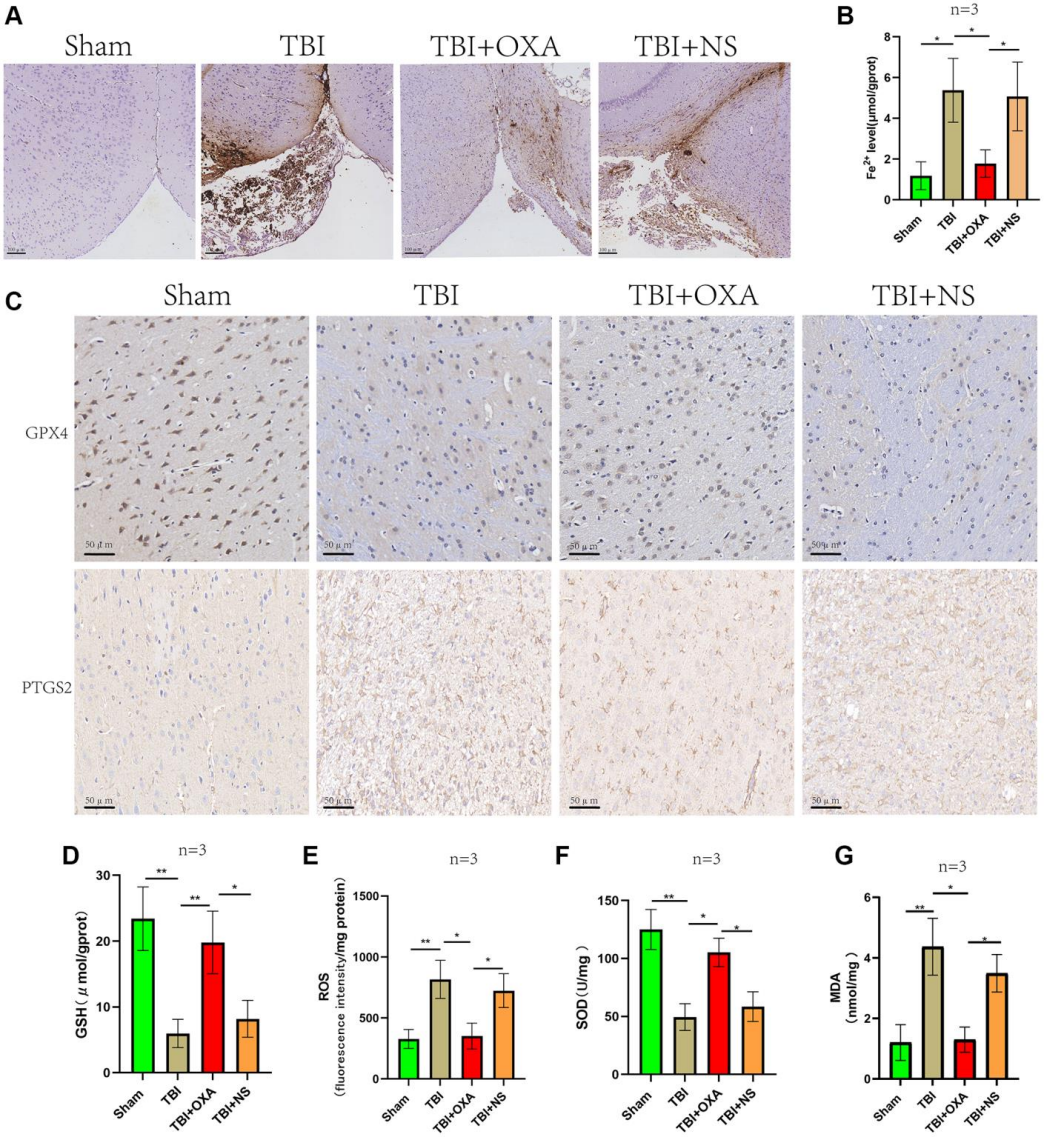
**OXA alleviates TBI induced Ferroptosis and oxidative stress.** (**A**) Perls staining showed iron deposition around the injured brain tissues of each rat group 24 hours after TBI. (**B**) Cortical non-heme iron was measured in sham, TBI, TBI+OXA, and TBI+NS groups. (**C**) Representative immunohistochemical staining of GPX4 and PTGS2. (**D**–**G**) Biochemical analysis was used to assess contents of GSH, SOD, MDA, and ROS in injured peripheral cortex of each group 24 hours following TBI. Findings are shown as mean ± standard deviation. ^*^P < 0.05, ^**^P < 0.01, ^***^P < 0.001, ^ns^*p* > 0.05.

**Figure 4:**
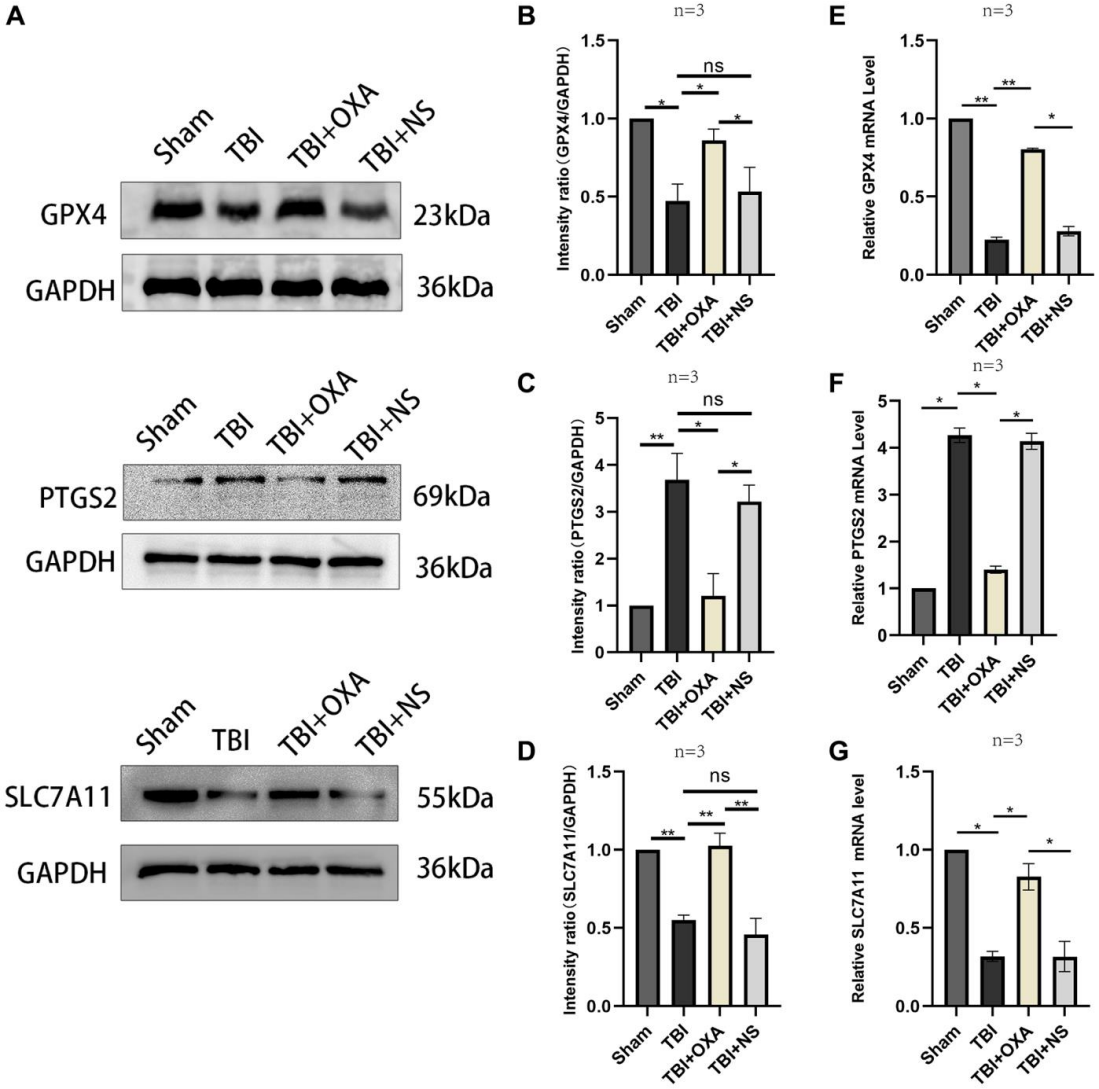
**Changes in markers related to ferroptosis following OXA treatment.** (**A**) Expression of GPX4, SLC7A11, and PTGS2 in injured peripheral cortex of rats following TBI was found via western blotting. (**B**–**D**) Western blot quantitative data for different groups. (**E**–**G**) Relative mRNA levels of GPX4, SLC7A11, and PTGS2 in injured peripheral cortex of rats in each group 24 hours after TBI. Findings are shown as mean ± standard deviation. ^*^P < 0.05, ^**^P < 0.01, ^***^P < 0.001.

First, we explored the effect of OXA on the markers of ferroptosis in the sham group. It was found that there was no significant change in the ferroptosis marker proteins in the sham group after intervention with OXA. (GPX4: F (2, 2) = 17.75, *P* = 0.1067; SLC7A11: F (2, 2) = 13.58, *P* = 0.1371, Figure 2A–2C). To determine whether OXA can rescue TBI-induced ferroptosis, we conducted Perls staining on tissue slices 24 h after TBI and determined iron content in cortex via tissue iron assay kit. Perls staining showed iron deposition around the injured brain tissues (Figure 3A). The findings revealed number of iron-positive cells observed in OXA treatment group was less relative to TBI and saline treatment groups (Fe^2+^: F (3, 8) = 9.161, *P* < 0.05, Figure 3B). In addition, immunohistochemical staining revealed relative to TBI and saline treatment groups, OXA treatment diminished PTGS2 expression and raised GPX4 expression in cortex 24 h following TBI (Figure 3C). Excessive lipid peroxidation, the primary biological characteristic of ferroptosis, has a close relationship with oxidative stress injury as well as ROS production. Therefore, we used biochemical analysis to evaluate the levels of MDA, GSH, SOD, and ROS in TBI rats’ brain tissue. Findings revealed relative to sham group, TBI and saline treatment groups exhibited significantly raised levels of MDA and ROS but significantly diminished levels of GSH and total SOD activity, indicating significant oxidative stress damages to brain tissue. OXA could reverse these changes (GSH: F (3, 8) = 15, *P* < 0.01, ROS: F (3, 8) = 12, *P* < 0.01, SOD: F (3, 8) = 21.33, *P* < 0.01, MDA: F (3, 8) = 16, *P* < 0.01, Figure 3D–3G), indicating that OXA could effectively protect brain tissue from oxidative stress damage after TBI. Next, we continued to measure the protein and mRNA levels of several ferroptosis biomarkers through western blotting and qPCR, including SLC7A11, PTGS2, and GPX4. Findings of western blot analysis coincided with qPCR. We found that relative to sham group, TBI and TBI+NS groups exhibited significantly increased mRNA and protein levels of PTGS2 but significantly decreased those of SLC7A11 and GPX4 at 24 h (protein:GPX4: F (3, 8) = 17.35, *P* < 0.001; SLC7A11: F (3, 8) = 24.62, *P* < 0.001; PTGS2: F (3, 8) = 11.95, *P* < 0.001; mRNA: GPX4: F (3, 8) = 53, *P* < 0.001; SLC7A11: F (3, 8) = 12, *P* < 0.001; PTGS2: F (3, 8) = 47, *P* < 0.001, Figure 4A–4G). OXA administration after TBI reversed these changes. In addition, to compare the effects of OXA and ferroptosis inhibitor, we used Lip-1 to intervene in TBI rats. The results showed that there was no significant difference in the inhibitory effect of OXA and Lip-1 on ferroptosis (SLC7A11, F (4, 10) = 82.23, *P* < 0.001; PTGS2, F (4, 10) = 11.05, *P* < 0.01; Figure 2D–2F). To further compare the protective effects of OXA and Lip-1 on the brain, we performed HE staining and found that the protective effect of OXA was better than that of Lip-1 (Figure 2G). In conclusion, above discoveries advice TBI results in changes in biomarkers of ferroptosis in injured peripheral cortex, while OXA treatment can effectively alleviate TBI-induced ferroptosis.

### OXA alleviates ferroptosis in TBI rat models via activating the Nrf2/HO-1 pathway

**Figure 5:**
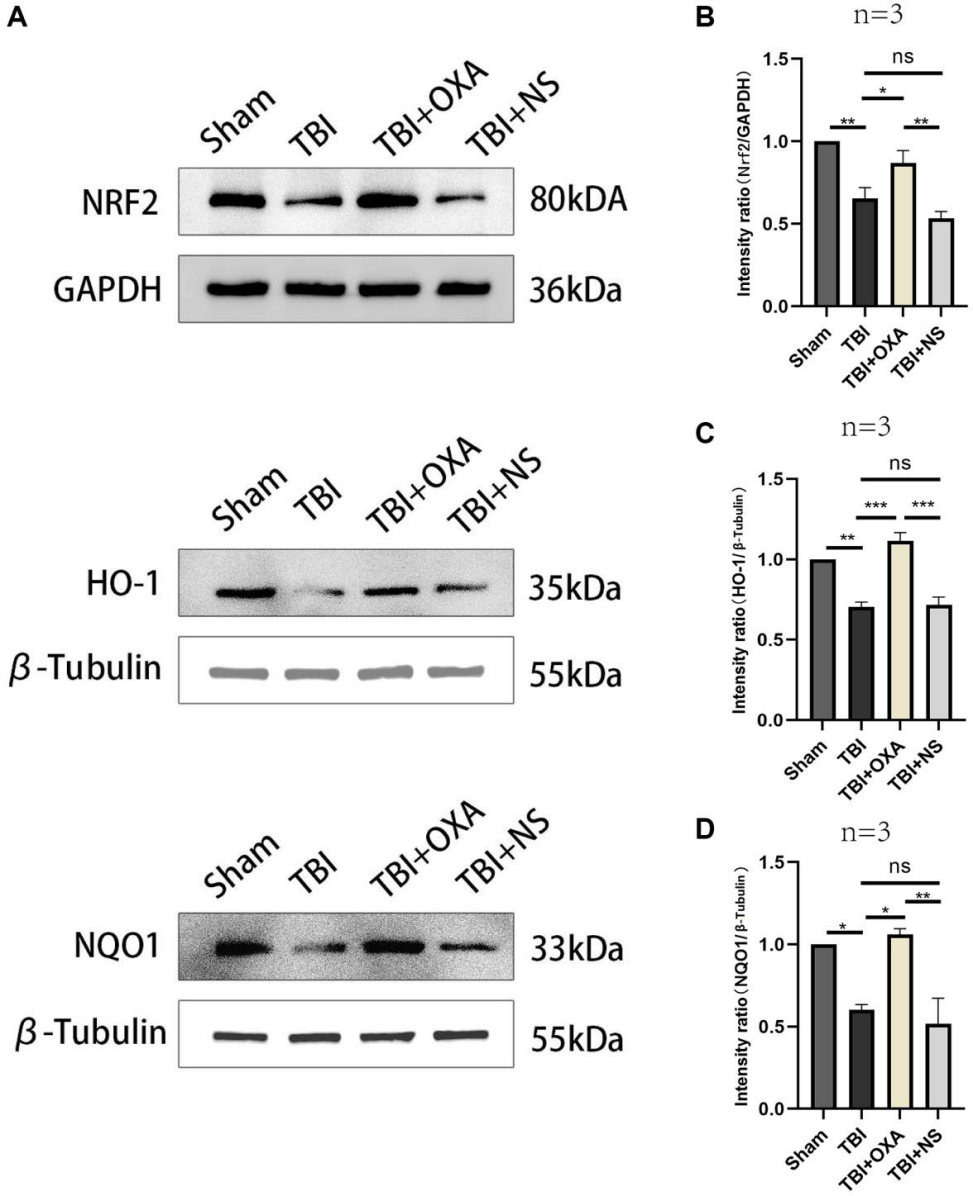
**OXA influences on Nrf2/HO-1 signal pathway in injured peripheral cortex in TBI model rats.** (**A**) Typical western blot bands. (**B**–**D**) Quantification of relative expression levels of HO-1, NRF2 and NQO1. Statistical data are shown in mean ± SEM. ^*^P < 0.05, ^**^P < 0.01, ^***^P < 0.001.

Nrf2/HO-1 is main signaling pathway in ferroptosis regulation. For elucidating possible mechanism of OXA’s protective functions, western blot analysis was utilized for measuring relative protein expression of Nrf2/HO-1 signaling pathway in surrounding tissues of the injury 24 h after TBI. Our results revealed relative to those in sham group, levels of Nrf2, NQO1, and HO-1 significantly diminished 24 h after TBI, and there existed nongreat difference between TBI and TBI+NS groups. OXA treatment increased expression of Nrf2, HO-1, and NQO1 after TBI (Nrf2: F (3, 8) = 19.89, *P* < 0.001; NQO1: F (3, 8) = 21.77, *P* < 0.001; HO-1: F (3, 8) = 18.66, *P* < 0.001; Figure 5A–5D). In addition, to further demonstrate that OXA relies on the Nrf2/HO-1 signaling pathway to alleviate ferroptosis, we also used the Nrf2 inhibitor ML385 to intervene in TBI rats. The results showed that the effects of both OXA and Lip-1 could be blocked by ML385, but ML385 had a stronger blocking ability on Lip-1 (SLC7A11, F (4, 10) = 82.23, *P* < 0.001; PTGS2, F (4, 10) = 11.05, *P* < 0.01; Figure 2D–2F). Therefore, we concluded that OXA may restrain ferroptosis through Nrf2/HO-1 signaling pathway activation.

### OXA improves functional outcomes and decreases lesion volume and rescues neuronal damage in the TBI mouse model

**Figure 6:**
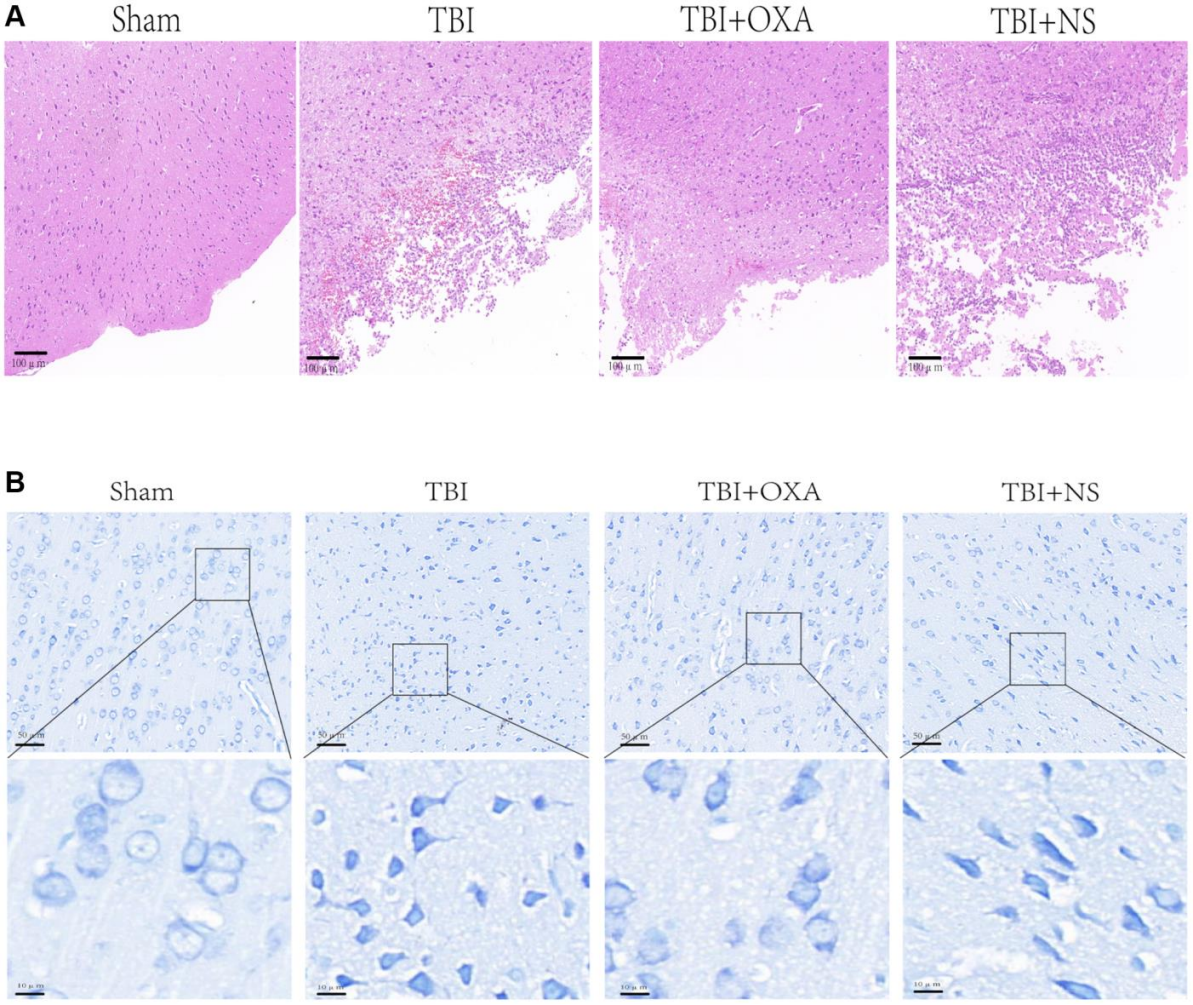
**OXA can alleviate brain tissue damage after TBI.** (**A**) Typical HE staining images of cortex in TBI rats of each group. (**B**) Representative Nissl stained images of cortex in TBI rats of each group.

**Figure 7:**
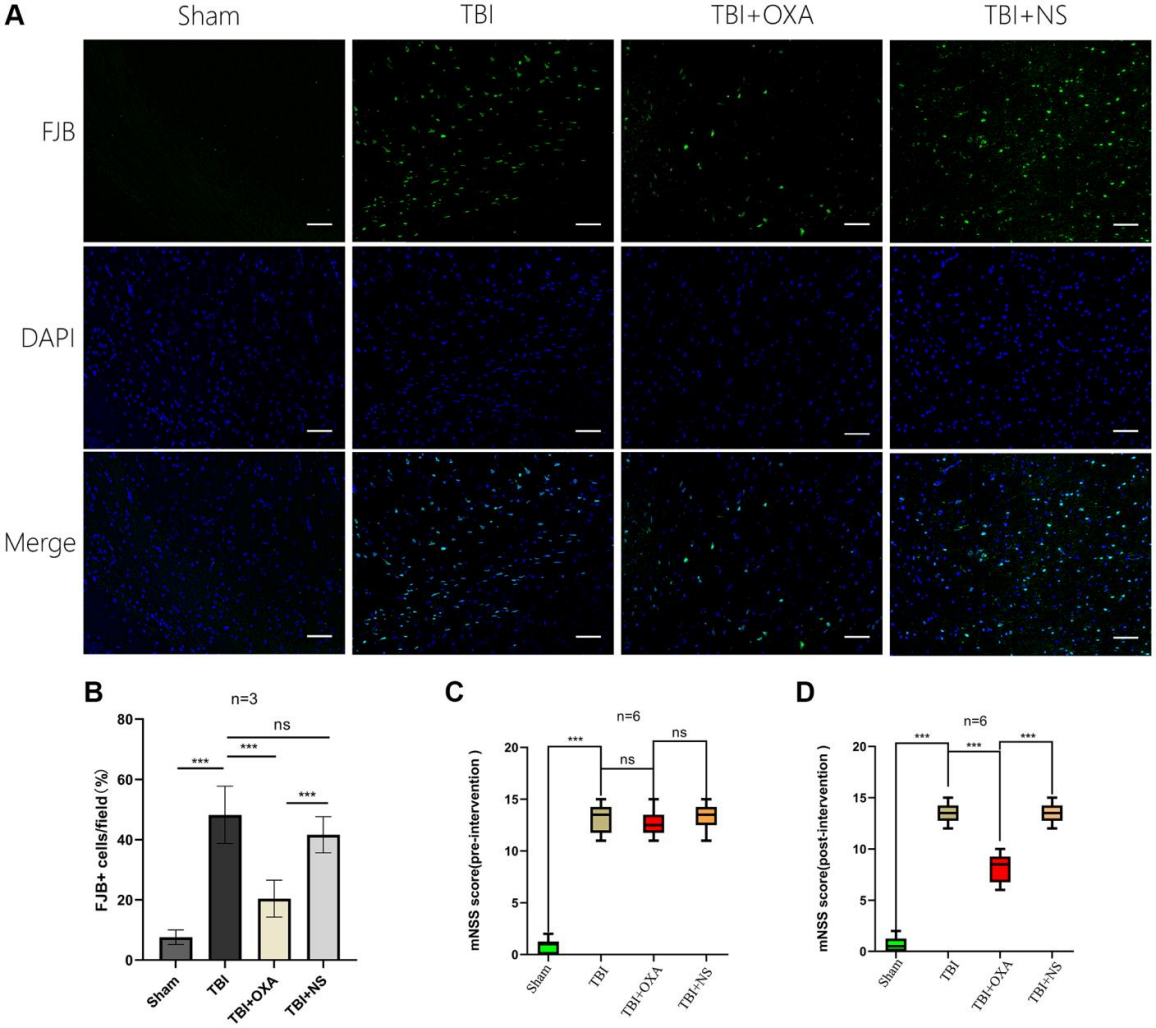
**OXA inhibits neuronal degeneration in the contused peripheral cortex after TBI and improves neural function after TBI.** (**A**) Typical images of Fluoro Jade B (FJB) staining of injured peripheral cortex 24 hours after injury. (**B**) Quantitative analysis showed number of FJB positive cells in TBI group remained greater in contrast with sham operation group. OXA effectively reduces number of degenerated neurons in contusion surrounding cortex following TBI. Data are shown as mean ± standard deviation. (**C**) Neurological functions were analysed via mNSS before treatment with OXA. There was no significant difference in mNSS scores between the TBI, TBI+OXA and TBI+NS group. Data are shown as median (P25, P75). (**D**) Neurological functions were analysed via mNSS 24 hours following TBI. Score in either TBI or TBI+NS group was tremendously greater relative to sham operation group, and OXA treatment reduced neurological deficit score. Data are shown as median (P25, P75), ^*^p < 0.05, ^**^p < 0.01, ^***^p < 0.001, ^ns^*p* > 0.05.

For farther studying OXA function in neuronal injury after TBI, HE, Nissl, and fluoro-Jade B (FJB) stainings were done. Discoveries of HE staining showed brain tissue in the sham group exhibited rich neuronal structures, with intact cell morphology and structure, a close arrangement between cells, and normal cell volume. In TBI and TBI+NS groups, structure remained loose, inflammatory cells were significantly infiltrated, and extensive vacuolar changes were observed. However, OXA treatment significantly improved these conditions (Figure 6A). Nissl and FJB staining were used to observe neuronal injury. Relative to sham group, TBI and TBI+NS groups displayed cytoplasmic contraction or nuclear pyknosis, and OXA treatment attenuated these histopathological changes (Figure 6B). Brain slices were stained by FJB for determining the neuronal degeneration 24 h following TBI, and it was discovered TBI increased number of degenerated neurons in cerebral cortex, while OXA greatly reduced number of degenerated neurons (F (3, 8) = 25.05, *P* < 0.001, Figure 7A, 7B). These data suggest that OXA can prevent TBI-induced ipsilateral cortical neuron damage. In addition, we performed the mNSS test for evaluating OXA influence on neurological deficits before treatment with OXA and 24 h following TBI. Before treatment with OXA, there was no significant difference in mNSS scores between the groups (Figure 7C). At 24 hours after TBI, relative to sham group, TBI group owned greater score and difference between TBI and TBI+NS groups was not found. However, OXA treatment significantly reduced the mNSS score, indicating that OXA owns protection influence on neuronal injuries (Figure 7D).

## DISCUSSION

Recently, research has found OXA and its receptors are vital for pathogenesis of neurological diseases like depression, narcolepsy, ischemic stroke as well as Alzheimer’s disease, and has attracted extensive attention. For our research, ferroptosis influence of OXA in TBI was studied. Major discoveries were that in acute phase of TBI, OXA treatment diminished brain tissue damages, improved neuronal death and improved short-term neurological function. We also found that OXA significantly activated Nrf2/HO-1 pathway in cortex of TBI rats. It remains initial study on elucidating protective influence of OXA on TBI-induced ferroptosis.

Ferroptosis, a new-found type of iron-dependent programmed cell death, is not the same as necrosis, apoptosis and autophagy. It was first discovered in tumor cells, but it has now been shown to be of significant importance in some neurological diseases like hemorrhagic and ischemic stroke, Parkinson’s and Alzheimer’s diseases [26, 27]. At the same time, research revealed ferroptosis has a close relationship with progression of cerebral cortex injury after TBI. Neuroprotective influence of preventing/restraining ferroptosis process is confirmed in TBI model [28–30]. Hence, identifying clinical medicines that can efficiently inhibit the process is indispensable. Previous research by our group found that non-invasive nerve-regulation technology can induce the increase of OXA in the cortex of TBI rats, reduce neuroinflammation, and play a neuroprotective role. What’s more, research revealed OXA can diminish neuronal damage via autophagy and apoptosis inhibition [15, 31–33]. However, the relation of OXA to ferroptosis with TBI conditions was not confirmed. In our research, we revealed inhibition influence of OXA on ferroptosis after TBI would be another action mode for OXA to exert neuroprotective effects.

Ferroptosis is triggered by ROS accumulation, GSH depletion, and GPX4 activity reduction and loss, which triggers programmed cell death [34]. Further studies show that an increase of PTGS2 expression level is another related molecular marker of lipid peroxidation during GPX4 inactivation-induced ferroptosis [35]. What’s more, the synthesis of GSH is regulated by cystine/glutamate transporter (system xc–), and SLC7A11, key factor in glutamate transport system, is vital for iron metabolism pathway. SLC7A11 level diminishment is able to reduce GSH level, causing GPX4 inactivation, and leading to ferroptosis [36–38]. We found expression of GPX4 and SLC7A11 diminished and PTGS2 level raised in cortex around the brain injury in rats at early stages following TBI. OXA treatment can partially reverse and improve the corresponding expression trends. Lip-1 is an inhibitor of ferroptosis, which prevents the accumulation of ROS and may play a neuroprotective role in acute central nervous system injury [39]. In our study, we compared the effects of OXA and Lip-1 in TBI rats. We found that OXA had similar ability to inhibit ferroptosis as Lip-1, but the neuroprotective effect of OXA was better than that of Lip-1. These results suggest that the neuroprotective effect of OXA in TBI rats is at least partially mediated by its ability to inhibit ferroptosis. MDA is one biomarker of lipid peroxidation and oxidative protein damage whereas SOD, GSH and GPX4 are endogenous antioxidant factors that are important reducing components that maintain homeostasis of the cellular redox environment [40, 41]. Further, with biochemical analysis we found that levels of iron and MDA in cortex around the TBI in rats raised whereas GSH content and total SOD activity decreased greatly, indicating a rise in lipid peroxidation levels in cortical tissue of TBI rat. OXA treatment could partially reverse and improve the corresponding changes. In addition, we also found that OXA treatment reduced TBI-induced brain tissue damage and motor dysfunctions through behavioral tests, HE staining, and Nissl staining. Above discoveries indicate OXA treatment can greatly diminish the lipid peroxidation level of injured cortex, inhibit ferroptosis, and increase the tissue-reducing capacity to resist oxidative stress damage.

Nrf2/HO-1/NQO1 pathway is vital for antioxidant stress response. Nrf2 is primary transcription factor for antioxidant stress response regulation, and HO-1 and NQO1 are primary downstream enzymes [42, 43]. Currently, Nrf2 is considered to be an important negative regulator in the process of ferroptosis [44]. Studies have also confirmed Nrf2 signaling pathway activation can induce expression of SLC7A11 and GPX4 of GSH antioxidant system, restrain ferroptosis and reduce neuronal loss [45, 46]. We discovered relative to sham group, levels of Nrf2 and downstream antioxidant proteins HO-1 and NQO1 in the injured cortex in TBI rat were greatly diminished whereas OXA treatment significantly raised the relevant expression. We also found that when we used ML385 to inhibit Nrf2, the ability of OXA and Lip-1 to resist ferroptosis was weakened, and ML385 had a stronger inhibitory effect on Lip-1 function. Our research results suggest that the ability of OXA and Lip-1 to resist ferroptosis is related to the activation of the Nrf2-ARE signaling pathway, but Lip-1 is more dependent on the Nrf2-ARE signaling pathway. Therefore, exploring more signaling pathways related to OXA’s ability to alleviate ferroptosis will also be our next focus. The above discoveries advise OXA treatment might diminish TBI-induced lipid peroxidation and restrain ferroptosis via Nrf2-ARE signaling pathway activation for neuronal damage and loss reduction. Based on these results, this study initially investigates the possibility of OXA exerting neuroprotective influences via oxidative stress and ferroptosis alleviation in neurons after TBI through the NRF2-ARE signaling pathway.

Our research has several limitations. Firstly, we merely investigated neuroprotective influence of OXA in early brain injury following TBI without evaluating its long-term performance on TBI. Therefore, research on the effect of OXA on chronic brain injury stage after TBI is required. Secondly, characteristics like gender, age, weight, species may be related to TBI prognosis; however, we merely investigated OXA influence on SD rats with analogous gender, age and weight. Thirdly, this study only evaluated the anti-ferroptosis effect of OXA and did not further investigate its impact on other mechanisms. There are further possible mechanisms that need to be investigated further.
